# New Computer-Aided Diagnosis of Dementia Using Positron Emission Tomography: Brain Regional Sensitivity-Mapping Method

**DOI:** 10.1371/journal.pone.0025033

**Published:** 2011-09-26

**Authors:** Akihiro Kakimoto, Yuichi Kamekawa, Shigeru Ito, Etsuji Yoshikawa, Hiroyuki Okada, Sadahiko Nishizawa, Satoshi Minoshima, Yasuomi Ouchi

**Affiliations:** 1 PET Medical Application Group, Central Research Laboratory, Hamamatsu Photonics K.K., Branch 5000, Hamamatsu, Japan; 2 Hamamatsu Medical Imaging Center, Hamamatsu Medical Photonics Foundation, Branch 5000, Hamamatsu, Japan; 3 Image-Guided Bio-Molecular Interventions Section, Department of Radiology, University of Washington School of Medicine, Seattle, Washington, United States of America; 4 Department of Biofunctional Imaging, Medical Photonics Research Center, Hamamatsu University School of Medicine, Hamamatsu, Japan; Federal University of Rio de Janeiro, Brazil

## Abstract

**Purpose:**

We devised a new computer-aided diagnosis method to segregate dementia using one estimated index (Total Z score) derived from the Brodmann area (BA) sensitivity map on the stereotaxic brain atlas. The purpose of this study is to investigate its accuracy to differentiate patients with Alzheimer's disease (AD) or mild cognitive impairment (MCI) from normal adults (NL).

**Methods:**

We studied 101 adults (NL: 40, AD: 37, MCI: 24) who underwent ^18^FDG positron emission tomography (PET) measurement. We divided NL and AD groups into two categories: a training group with (Category A) and a test group without (Category B) clinical information. In Category A, we estimated sensitivity by comparing the standard uptake value per BA (SUVR) between NL and AD groups. Then, we calculated a summated index (Total Z score) by utilizing the sensitivity-distribution maps and each BA z-score to segregate AD patterns. To confirm the validity of this method, we examined the accuracy in Category B. Finally, we applied this method to MCI patients.

**Results:**

In Category A, we found that the sensitivity and specificity of differentiation between NL and AD were all 100%. In Category B, those were 100% and 95%, respectively. Furthermore, we found this method attained 88% to differentiate AD-converters from non-converters in MCI group.

**Conclusions:**

The present automated computer-aided evaluation method based on a single estimated index provided good accuracy for differential diagnosis of AD and MCI. This good differentiation power suggests its usefulness not only for dementia diagnosis but also in a longitudinal study.

## Introduction

The number of patients with dementia in the world is increasing every year [1]. Specifically, Alzheimer's disease (AD) and mild cognitive impairment (MCI) are worth noticing because AD accounts for 60% of the dementia population and the probability of MCI progression to AD is considered 11 to 33% in two years [2]. On the bright side, a number of promising therapeutic measures against dementia are under way [3]–[6], which then brings the idea that early detection and accurate differentiation are of great importance. Examination procedures to promote early detection and facilitate an accurate differential diagnosis include diagnostic imaging procedures, such as positron emission computed tomography (PET), single photon emission computed tomography (SPECT), and magnetic resonance imaging (MRI). In particular, ^18^FDG PET is useful in patients under a tentative diagnosis of degenerative brain disease and in early detection of dementia [7], [8]. Although imaging technical advances such as in vivo visualization of a pathological substance amyloid protein are now available in AD detection, the usefulness of ^18^FDG PET, which facilitates early diagnosis based on the pattern of altered brain metabolism, is still emphasized [9]–[19].

There are many computer-aided diagnosis (CAD) tools for detection of dementia. Among them, 3D-SSP (NEUROSTAT) is a widely-used imaging tool in the clinical setting [20] in contrast to statistical parametric mapping (SPM) as rather a research tool [21] for evaluating the rate of reduction in comparison with normal group. In particular, 3D-SSP excels in visual assessment of metabolic changes in the brain. However, when investigating serial changes in the same patient or therapeutic intervention-related changes, a more objective analytical method is preferable and elimination of subjective diagnostic factors such as visual searching or manipulation of region selection is necessary.

Thus, we aimed to differentiate AD patients from normal subjects or MCI patients using a new CAD method automatically. To this end, we first determined 34 BA regions on projected images of the brain surface in reference to the BA map [22], and generated sensitivity-distribution maps to compare the standard uptake value ratio (SUVR) in each brain region among the NL and AD groups. Finally, we verified the segregation power of this method by applying it to the MCI group.

## Materials and Methods

### Subjects

The current study was approved by the Ethics Committee of Hamamatsu Medical Center, and written informed consent was obtained from each participant after detail explanation of this study. We performed PET measurements with ^18^FDG for all participants (n = 101) and used their ^18^FDG images for the current purpose. They consisted of 40 normal volunteers (NL) (18 males, 22 females, mean age: 55.8±17.1 years) with normal MR findings and normal cognition by mini-mental state examination (MMSE) [23], 37 patients with AD (13 males, 24 females, mean age: 59.4±6.6 years) diagnosed on the basis of the NINDS-ADRDA [24] and DSM-IV [25] criteria, and 24 patients with MCI (9 males, 15 females, mean age: 69.2±9.9 years), who met Peterson's criteria for amnestic MCI [26]. All MCI patients were annually evaluated clinically for 3 years, and 10 amnestic MCI patients (3 males, 7 females) were converted as AD (called as an AD-converter) and other 14 patients (6 males, 8 females) remained amnestic MCI (called as a non-converter).

Using the SPSS (Version 17.0) Random Number Generator Tool, the two groups (NL and AD) were arbitrarily divided into two categories (Table 1). We confirmed that there were no significant differences in the age, sex, or MMSE scores between the two categories (p>0.1). One was a training group for generation of a sensitivity distribution index (Category A), and the other was a test group (Category B) for verification of the index obtained from Category A.

**Table 1:** Subject characteristics.

Group	NL		AD		MCI	
Category	A	B	A	B	AD-converters	Non-converters
Number	20	20	18	19	10	14
Male/Female	9/11	9/11	6/12	7/12	3/7	6/8
Age (years)	56.0±15.4	55.7±19.1	59.4±7.5	59.3±5.7	64.5±9.5	72.6±9.0
MMSE* (score)	29.0±1.1	29.1±1.1	16.7±5.4	16.5±5.1	23.7±3.4	26.6±1.4

*MMSE = mini-mental state examination.

### ^18^FDG PET scanning

We used an SHR-12000 Brain PET camera (Hamamatsu Photonics K.K.) with intrinsic resolution, 2.9×2.9×3.4 mm full-width half-maximum (FWHM), 47 slices obtained simultaneously, and 163 mm axial field of view [27]. After transmission scan for attenuation correction was performed for 10 minutes, 1.5 MBq/kg of ^18^FDG was injected through the cubital vein. After each subject rested in a dimly-lit room for 45 minutes, emission was measured in 2D mode for 15 minutes. The filtered back projection (FBP) method was employed for image reconstruction. The matrix and pixel size of the reconstructed image were 192×192×47 and 1.3×1.3×3.4 mm, respectively.

### Demarcation of the Brodmann area

**Figure 1:**
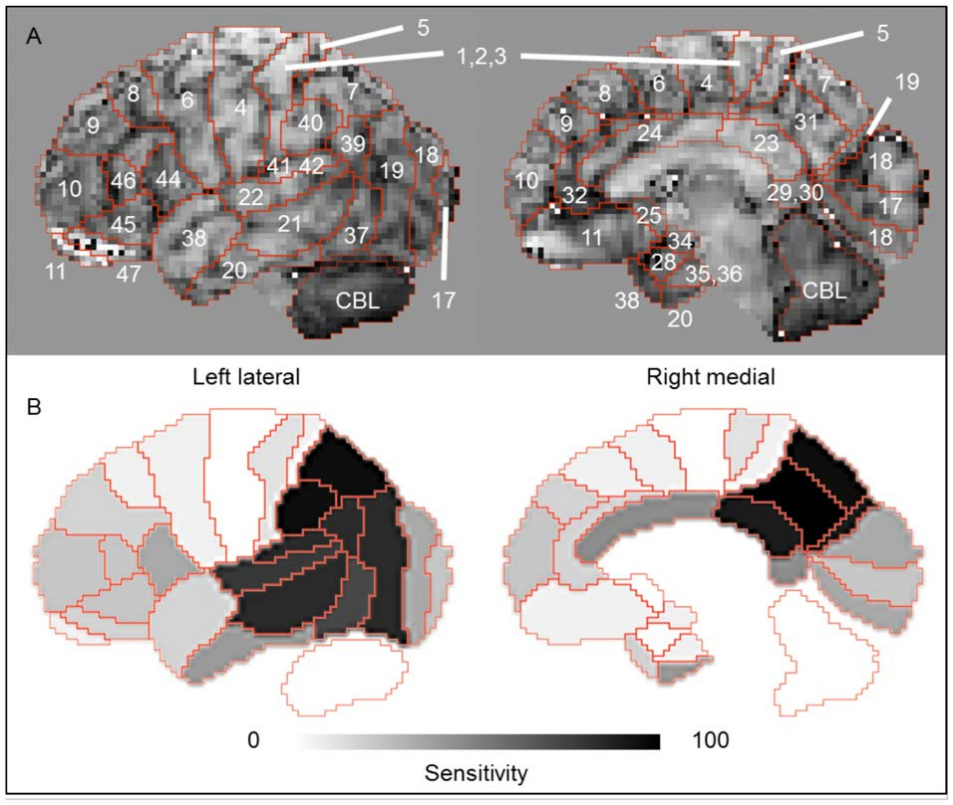
BA on 3D-SSP images. BA on 3D-SSP MRI template images (A) and sensitivity-distribution maps of BA on 3D-SSP images among NL and AD in Category A (B). The color bar denotes the levels of sensitivity to differentiate NL and AD in Category A.

On the brain surface projection atlas (MRI template) from the 3D-SSP tools [20], 34 regions were determined as Brodmann areas (BAs) [22] by 1 neurologist and 2 radiological technicians (Fig. 1A). In demarcation of BAs, by referring to the Talairach Atlas [28] which describes BAs along with the names of gyri, we reconstructed the BA fields in the axial direction and were able to allocate BAs on the surface of lateral and medial view of the spatially normalized brain. In definition, BAs consist of Areas 1 to 52, which are categorized on the basis of neuronal structure in the cerebral cortex stained in the postmortem brain. In the present study, we unified BAs 1–3, 29–30, 35–36, and 41–42 as each single area due to the small pixel count. We had to exclude BA 12, 13, 14, 15, 16, 26, 27, 33, 43, 48, 49, 50, 51, and 52 because of no visualization on the brain lateral projection surface. By adding the cerebellum, a total of 34 regions were determined in this study. Because the characteristic pattern of ^18^FDG accumulation in dementia on 3D-SSP map is highlighted in the lateral views [9]–[14], we excluded the anterior/posterior/superior/inferior views. As the right-left difference in ^18^FDG accumulation is not a critical matter in the AD diagnosis, the bilateral values were treated as a mean single value in the present study.

### Image analysis

Using a 3D-SSP anatomical standardization tool, ^18^FDG PET images were normalized to Talairach's standard brain images [28]. Subsequently, peripheral noises outside the brain were removed using standard brain mask images, and the mean whole-brain pixel count in the standard brain (standard uptake value (SUV)) was calculated. Based on the mean SUV for the standard brain in the NL group in Category A, each pixel was corrected as the SUV ratio (SUVR) by dividing all pixels by SUV of the whole brain. Some researchers assume the pons as an area of the reference for SUV normalization in 3D-SSP. However, the global mean is considered as a better index for the normalization than the pons due to the less occurrence of misregistration of ROI on the small area pons. The accuracy of our method was dependent on the value in the NL group, in which the smaller variation of mean value was more important. Therefore, the whole brain was chosen as the region for correction of the count normalization.

On projected images of the brain surface, we determined the mean SUVR for each BA, and then calculated the mean SUVR and standard deviation per BA in the NL group in Category A. Using these values and equation (1), the SUVR was converted to the Z-score per BA (Z_NL_n_, n = Area number); here, Z-score per area (not Z-score per pixel) was calculated.
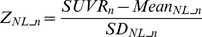
(Eq.1)SUVRn indicates the SUVR for Area n in a subject. The Mean_NL_n_ and SD_NL_n_ were the mean SUVR and standard deviation in the NL group of Category A, respectively.

### Sensitivity

In Category A, the Z_NL_n_ values for each BA were compared between NL and AD subjects. We calculated sensitivity using a cut-off value determined from SUVRs for the NL group of Category A by measuring the amount of SUVR values lower than the cut-off in the AD group. The cut-off value was determined as Z_NL_n_ = −1.0, because the proportion of Z_NL_n_ values of -1.0 or more in all values was 0.8413 in the standard normal distribution table; the specificity of NL assessment may be fixed at approximately 80%. The value -1.0 as a cut-off level was determined through preliminary experiments, in which changes in cut-off value caused to affect the final accuracy. The value −1.0 was found good enough to guarantee above 80% specificity considering the Z-score distribution. The sensitivity per BA, which reflects the diagnostic capacity for AD, was calculated (W_NL-AD_1_ to W_NL-AD_34_).

### Total Z-score method

In practice, no radiologist would diagnose diseases only by looking at an abnormality of a single domain of the brain. Instead, they dedicate themselves to visual searching for every part of the brain and make a judgment through the comprehensive inspection. To eliminate this laborious work, in this study, we devised the Total Z-score method, which allows physicians to make a diagnosis based on the comprehensive assessment of all areas without focusing on each BA.

First, we estimated the weighted sensitivity per area. By inserting this value and each subject's Z-score into the following equation (2), all areas may be comprehensively evaluated based on a single value. As Sum_NL-AD_ is multiplied by the sensitivity value per BA, the site of disease-specific reduction may be emphasized, improving the diagnostic capacity:
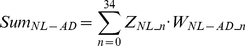
(Eq.2)Z_NL_n_ indicates the Z-score for Area n in a subject. It is based on data from the NL group in Category A. W_NL-AD_n_ refers to the sensitivity to differentiate AD from NL in Area n. In all subjects, values were inserted into the equation (2), and Sum_NL-AD_ was calculated in each subject.

Using equation (3), Sum_NL-AD_ was converted to the Z-score (Z_NL-AD_) based on the mean/standard deviation in the NL group of Category A. The Mean_NL-AD_ and SD_NL-AD_ were a mean value of Z_NL-AD_ and its standard deviation in the NL group of Category A, respectively.
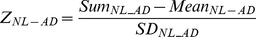
(Eq.3)Thus, the Total Z-score, which reflects the comprehensive evaluation of the SUVRs or 34 BAs on brain surface projections, Z_NL-AD_ was calculated in each subject. A high Z_NL-AD_ value suggests an NL condition, whereas a low value suggests AD.

The value Sum_NL-AD_ was generated by adding 34 products of multiplication of Z_NL_n_ by W_NL-AD_n_, where the sensitivity W_NL-AD_n_ was used as a weighted index. For instance, in a brain region that clearly differs NL from AD, Z_NL_n_ in NL subjects is high while Z_NL_n_ in AD patients is low, resulting in the sensitivity W_NL-AD_n_ being high. Thus, the index W_NL-AD_n_ makes the difference of Sum_NL-AD_ between NL and AD more remarkable by weighting the value Z_NL_n_. In contrast, in an area with negligible difference between NL and AD, there is no significant gap in Z_NL_n_ of NL and AD, resulting in the sensitivity W_NL-AD_n_ being low. This makes the product (Z_NL_n_ × W_NL-AD_n_) much smaller. Then, the product Sum_NL-AD_ is converted to Z_NL-AD_ using Eq. 3. In this way, an initial determinant (a cut-off value) can differentiate groups by weighting values in each brain area.

### NL-AD differentiation

Using the Total Z-score (Z_NL-AD_), differential analysis of NL and AD was conducted to evaluate the accuracy. Before this differentiation was performed, we determined a cut-off value with which NL-AD pair was compared using the SPSS software (Version 17.0). We estimated a receiver operating characteristic curve (ROC) and the area under the curve (AUC) based on the Z_NL-AD_ values for the NL and AD groups in Category A. The most appropriate cut-off value (C_NL-AD_) was determined by the Youden index [29]–[31]. Based on C_NL-AD_, Categories A and B were classified into NL or AD, respectively, to evaluate the accuracy.

### Application to MCI

We applied this method using Z_NL-AD_ and C_NL-AD_ to ^18^FDG PET images of amnestic 24 MCI patients scanned at entry to verify the usefulness of this program in differentiation of AD from MCI.

## Results

### 3D-SSP Z-score images

**Figure 2:**
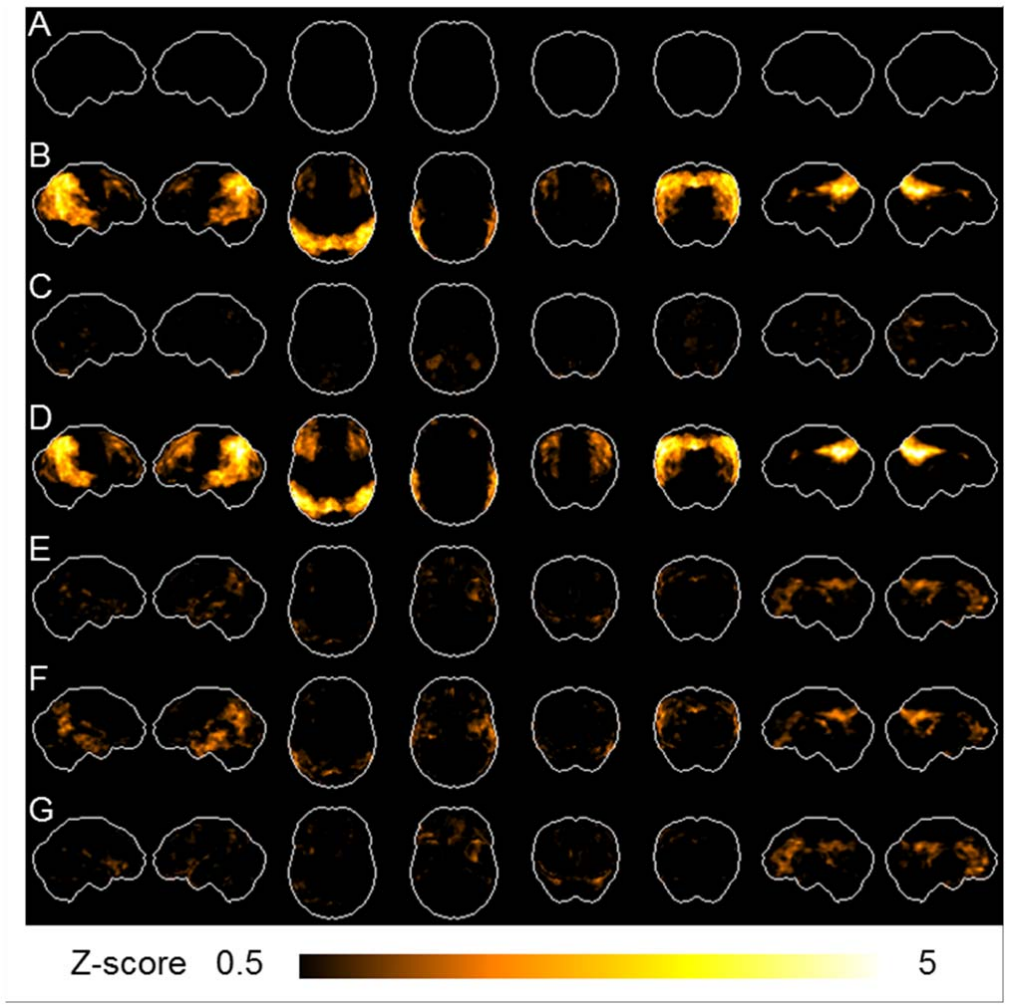
3D-SSP images. Employing 20 NL subjects in Category A as a reference database, all subjects' 3D-SSP Z-score images were prepared. Average Z-score images of 20 NL (A) and 18 AD (B) in Category A. Average Z-score images of 20 NL (C) and 19 AD (D) in Category B. Average Z-score images of a total of 24 MCI (E), 10 AD-converters (F) and 14 non-converters (G). The color bar denotes the levels of Z-score based on 20 NL subjects in Category A.

In the NL group in Category A, the mean SUV for the standard brain was 5.75. Employing these subjects as a reference database, all subjects' 3D-SSP Z-scores were calculated by pixel and average 3D-SSP Z-score images were prepared in 20 NL (Fig. 2A) and 18 AD (Fig. 2B) subjects in Category A, as well as in 20 NL (Fig. 2C) and 19 AD (Fig. 2D) subjects in Category B, respectively. As shown Figure 2, in the NL and AD groups, there was no marked difference between Categories A and B. In the NL group, there was no marked reduction in either group. In the AD group, there were marked decreases in the lateral parietal, lateral temporal and cingulate gyrus area. Additionally, average images were prepared in all 24 MCI (Fig. 2E), 10 AD-converter (Fig. 2F) and 14 non-converter (Fig. 2G), respectively. In the MCI patients, there were greater decreases of glucose metabolism in the lateral parietal, lateral temporal and medial parietal areas in AD-converters than non-converters

### BA-based analysis

The quantitative SUVRs in the NL and AD groups in Category A are shown in Table 2. There were significant differences in the SUVRs for BAs 7, 19, 21, 22, 23, 31, 37, 39, 40, and 41 (42) between the NL and AD groups (p<0.001). In the AD group, the mean SUVRs of each BA region were 20, 13, 15, 8, 23, 22, 16, 17, 17, and 10% lower than those in the NL group, respectively.

**Table 2:** SUVR and sensitivity of Brodmann area.

No.	Brodmannarea	SUVR*		Sensitivity (%)	No.	Brodmann area	SUVR		Sensitivity (%)
		NL	AD	W_NL-AD_n_			NL	AD	W_NL_AD_n_
1	1, 2, 3	6.81±0.30	7.25±0.51	11.1	18	25	6.88±0.28	7.49±0.64	0
2	4	6.99±0.25	7.64±0.48	0	19	28	3.44±0.29	3.93±0.29	0
3	5	7.03±0.48	7.38±0.61	5.6	20	29, 30	6.64±0.50	6.31±0.44	44.4
4	6	7.32±0.24	7.48±0.49	5.6	21	31	8.76±0.42	7.20±0.65	100
5	7	7.49±0.32	6.26±0.60	94.4	22	32	6.76±0.31	6.95±0.55	16.7
6	8	7.36±0.33	7.37±0.37	5.6	23	34	4.14±0.39	4.38±0.30	5.6
7	9	7.12±0.27	7.10±0.37	16.7	24	35, 36	4.63±0.29	4.84±0.39	5.6
8	10	7.02±0.26	7.13±0.47	22.2	25	37	7.20±0.24	6.21±0.73	72.2
9	11	6.60±0.27	7.02±0.53	5.6	26	38	5.76±0.20	5.80±0.31	16.7
10	17	8.03±0.60	8.01±0.65	22.2	27	39	7.31±0.27	6.24±0.67	83.3
11	18	7.59±0.38	7.40±0.60	33.3	28	40	7.04±0.23	6.01±0.48	94.4
12	19	8.75±0.36	7.75±0.61	88.9	29	41, 42	7.20±0.28	6.55±0.45	83.3
13	20	5.84±0.19	5.64±0.41	38.9	30	44	7.15±0.27	7.12±0.40	33.3
14	21	7.06±0.26	6.14±0.60	83.3	31	45	7.07±0.23	7.23±0.44	22.2
15	22	7.21±0.27	6.70±0.32	77.8	32	46	7.01±0.26	7.10±0.43	22.2
16	23	7.63±0.46	6.19±0.73	88.9	33	47	6.64±0.26	6.87±0.46	16.7
17	24	5.89±0.39	5.60±0.54	44.4	34	CBL**	5.99±0.33	6.74±0.48	0

*SUVR: standard uptake value ratio (mean ± SD).

**CBL: cerebellum.

As shown in Table 2 (sensitivity), the W_NL-AD_ value for BA 31 was the highest (100%), followed by 94.4% for BAs 7/40, 88.9% for BAs 19/23, and 83.3% for BAs 21/39/40(41). As illustrated in the Figure 2, the maps show the distribution of the sensitivity to differentiate NL from AD. Figure 1B shows the sensitivity of each BA with a gray scale, in which the black indicates higher sensitivity.

### Comparison between NL and AD groups

**Figure 3:**
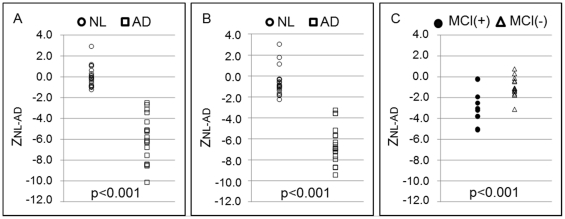
The dot plots of Total Z-score. The dot plots of Z_NL-AD_ in Category A (A), Z_NL-AD_ in Category B (B), and AD-converters (MCI+) and non-converters (MCI-) (C).

To perform the 2-group differential analysis of NL and AD, we estimated the cut-off value (C_NL-AD_) based on the Z_NL-AD_ values. First, we made the dot plots (Fig. 3A) of Z_NL-AD_ in the NL and AD groups in Category A, and the AUC was calculated to be 1.00. As a result, the most appropriate cut-off value (C_NL-AD_) was determined to be −1.9 by the Youden index. Furthermore, we evaluated the differentiation power by C_NL-AD_, and the sensitivity and specificity in Category A were found to be all 100%.

Using equations (1) to (3) and the sensitivity-distribution maps (Fig. 1B) based on SUVRs of NL and AD groups in Category A, Z_NL-AD_ of each subject in Category B were calculated (Fig. 3B). In Category B, the sensitivity and specificity were found to be 100% and 95%, respectively.

### Detection of AD in the MCI group

We made the dot plots of Z_NL-AD_ in 24 MCI patients who had been classified into two groups; AD-converters and non-converters diagnosed clinically during the 3-year follow-up period. Using the cut-off value determined in the Category A (C_NL-AD_), our program judged 9 patients as AD (38%) and 15 patients as NL (62%). During the 3-year follow-up, 10 patients were converted from MCI to AD (AD-converters) and the residual 14 MCI patients were still under the MCI condition. As shown in Figure 3C, 8 out of 10 AD-converters were determined as AD by our program (80%), and 2 out of 10 AD-converters as NL (20%). In contrast, 13 out of 14 non-converters were determined as NL by our program (93%), and 1 out of 14 as AD (7%). This yielded the sensitivity and specificity for differentiating AD-converters from non-converters in MCI patients by our CAD program to be 80% and 93%, respectively, with an accuracy of 88%.

## Discussion

In this study, we developed a new CAD analytic tool using BA compartmentalization on 3D-SSP atlas, and calculated the Total Z-score through the complex observation of all areas based on the sensitivity (weighted value) per area to investigate the differential accuracy of images. When employing this method, the sensitivity and specificity for differentiating AD from NL were all 100%, in the training group, with an accuracy of 100%. In the test group, they were found to be 100 and 95%, respectively, with an accuracy of 98%. Furthermore, the sensitivity and specificity for differentiating AD-converters from non-converters in patients with MCI were 80% and 93%, respectively, with an accuracy of 88%. As shown in Fig. 1 and Table 2, the sensitivity (W_NL-AD_n_) map showed characteristic patterns similar to the FDG patterns seen in AD in the previous literature [8]–[14]; hypometabolism in the parietal (BAs 7, 19, 39, and 40), temporal (BAs 21, 22, 37, and 41 (42)), and cingulate (BAs 7, 23, and 31) areas in patients with AD.

A CAD method is never new now, but the level of its accuracy is still a target of improvement. Previous CAD methods using both statistical mapping technique and ROI analysis reported about high accuracy for differentiating AD from NL [32], [33]. These methods used specific ROIs or combination of multiple ROIs to discriminate one group from another. In contrast, our method used all ROIs (BAs) to estimate one unified value as a Total Z-score that was the product of the sensitivity of each ROI. This method consisting of more objective and CAD-oriented algorism can eliminate any subjective errors and bias and enables more accurate and objective diagnosis than those other methods. Indeed, the present method generated 98% in accuracy for discriminating AD from NL.

This program affording a high segregation power was also shown to be effective to extract AD-like images from the group of MCI, resulting in good accuracy (88%) for differentiating AD-converters from non-converters. Previous CAD methods were reported to exhibit up to 90% in accuracy for differentiating AD-converters from non-converters [34]–[36] among MCI patients. However, ROI assessment embedded in their programs seemed less objective than our method. As shown in Figure 2, 3D-SSP provided visual presentations characteristic to AD-converters (Fig. 2F) and non-converters (Fig. 2G), where there were greater decreases of glucose metabolism in the lateral parietal (BAs 7, 39, and 40), lateral temporal (BAs 21 and 37), and medial (BAs 7 and 31) areas in AD-converters than non-converters. Our method enabling objective assessment using a Total Z-score value without visual inspection showed the BAs distribution similar to the high sensitivity areas in the sensitivity-distribution maps (Fig. 1B). It is worth noting that an MCI patient with a high chance of AD conversion would show such a hypometabolic pattern seen in those BAs. Although the conversion rate from MCI to AD was reported to be 11–33% [2], the rate (42%) in our study was shown to be higher possibly because the observation period for disease conversion was one year longer in our study. Because we did know who were converted as AD during the 3-year follow-up, we were able to calculate the sensitivity and accuracy of this method in differentiation of AD from MCI by comparing the number of program-based AD patients with that of clinically diagnosed AD-converters.

Several CAD methods were reported in the past. In the literature, they used a channelized Hotelling observer (CHO) method or a principal component analysis (PCA) after setting volume of interests (VOIs) for diagnosing AD or MCI. The merit of using CHO [37] is to differentiate patterns of frequency after Fourier transformation of levels of pixels measured by SPECT between groups. Using voxel data [37], [38] sounds more objective, but a high chance of noise generation may degrade the image quality. In contrast, the use of VOI that contains multiple pixels would improve the reliability of segregation. Some researchers used PCA for fixed ROIs determined a priori [39]–[41], where relatively lower sensitivity and specificity were reported than those of our study. One reason of our high accuracy may be the fact that all our ROI data were converted to the sensitivity values irrespective of regions of specificity, although PCA needs to select the region specific to the disease beforehand. Indeed, our preliminary data using PCA for our ROI data generated 5∼10% reduction in accuracy (data not shown). In addition, our CAD advantage is the flexibility in applying this method to any disease segregation because a priori ROI determination is unnecessary.

**Figure 4:**
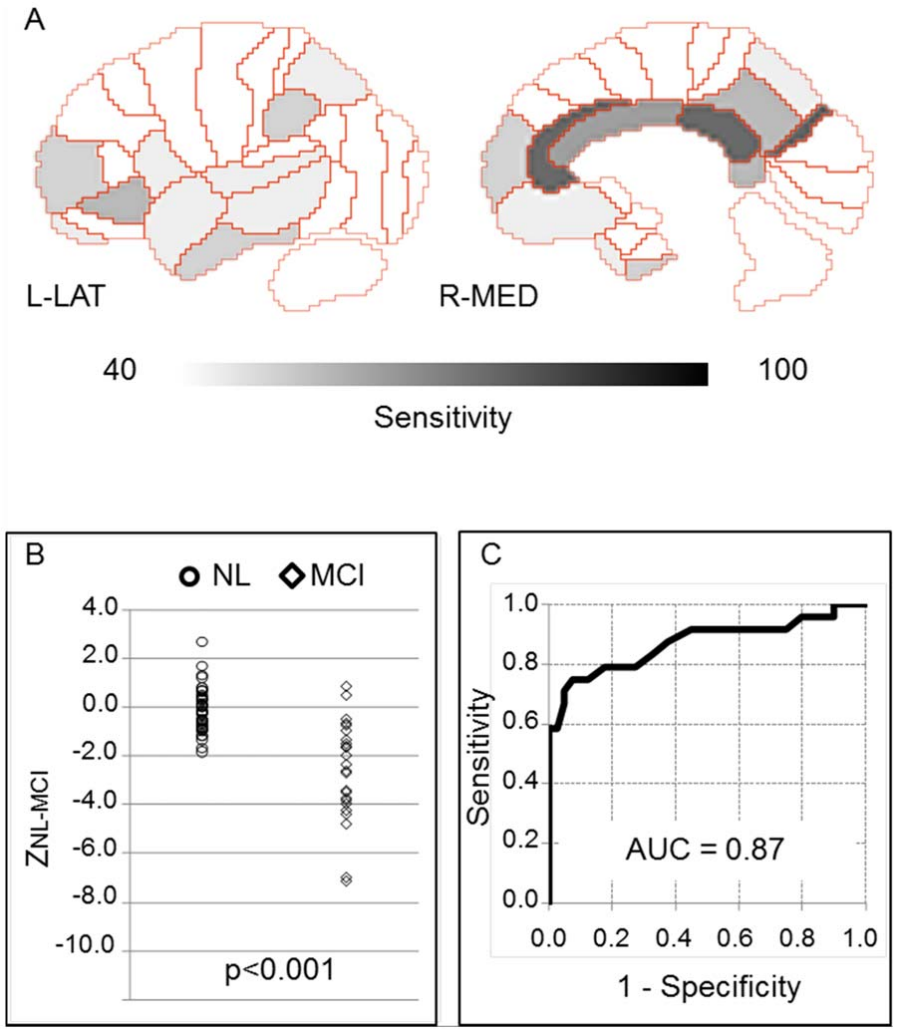
Sensitivity-distribution maps among NL and MCI. Sensitivity-distribution maps of BA (A), dot plots (B), and ROC (C) between NL and MCI. The color bar denotes the levels of sensitivity to differentiate NL in Category A and MCI groups.

There were methodological issues to be noted in our CAD method. Our program takes advantage of the patterns of regional sensitivity to differentiate AD from NL, and the generated sensitivity-distribution map (Fig. 1B) is a core of our method. Any core map cannot be complete, and a small variation of computer-generated sensitivity would lead to misdiagnosis. This kind of error may reflect intrinsic limitations of any automated imaging analyses including CAD technique because a pixel-value within a ROI has to be determined by a threshold. Therefore, although our method is useful and helpful in differential diagnosis of amnesic diseases, any CAD-induced outcomes should be accompanied with detailed clinical assessment to minimize misdiagnosis in the clinical setting. A good point of another issue is its versatility. In this study, our program is not designated as a tool for discriminating MCI from NL. If this segregation is a target, the Total Z-score Z_NL-MCI_ from the sensitivity-distribution maps among the NL and MCI groups may be appropriate. To evaluate the differentiation power of the Total Z-score Z_NL-MCI_, we made sensitivity-distribution maps (Fig. 4A) between NL in Category A and MCI group. Using these maps and Equation (1) to (3) by changing AD data into MCI data, we calculated the Total Z-score Z_NL-MCI_ (Fig. 4B). Employing the Youden index, cut-off values showing the most accurate differential diagnostic capacity was calculated: C_NL-MCI_ = −1.3. In addition, the area under the curve (AUC) value was 0.87 (Fig. 4C). In any case, this BA-based procedure has a potential to be applied for the differential diagnosis of many other brain diseases such as FTD and DLB with a specific pattern of neuronal degeneration.

In conclusion, our newly developed CAD method has a good power to discriminate AD from NL with an accuracy of 98%. This program also showed a good performance in detecting AD-converters among amnestic MCI patients with an accuracy of 88%. These results suggest the usefulness of this procedure for the differential diagnosis of AD/MCI as a diagnosis-assisting method free of any human judgment. Because the calculated Total Z-score is an objective value, our method with this index enables the semi-quantitative assessment of metabolic reduction and follow-up in dementia. This BA-based procedure can be also applied for the differential diagnosis of many other brain diseases such as FTD and DLB with a specific pattern of neuronal degeneration.
